# 114 Long Term Impact of Hospital Acquired Multi-drug Resistant Organisms on Health-related Quality of Life

**DOI:** 10.1093/jbcr/irac012.116

**Published:** 2022-03-23

**Authors:** Nathaniel K Ashford, Jamie Oh, Kara McMullen, Gretchen J Carrougher, Sean A Hickey, Colleen M Ryan, Jeffrey C Schneider, Nicole S Gibran, Barclay T Stewart

**Affiliations:** University of Washington, Seattle, Washington; University of Washington, Seattle, Washington; University of Washington, Portland, Oregon; Department of Surgery, The University of Washington, Seattle, Washington; Massachusetts General Hospital, Boston, Massachusetts; Harvard Medical School, Boston, Massachusetts; , Massachusetts; University of Washington, Wellfleet, Massachusetts; University of Washington, Seattle, Washington

## Abstract

**Introduction:**

MDROs colonize wounds and cause infections for hospitalized burn patients, which may lead to increased infection risk, wound complications, longer (LOS) and more cost. Little is known about the long-term impacts of MDRO colonization and infection on burn survivors. We aimed to describe the impacts of colonization on long-term health-related quality of life (HRQoL), itch, and pain.

**Methods:**

Data from adult participants in a multicenter longitudinal outcome study were used. Data was described and χ ^2^ and Kruskal-Wallis testing was applied to determine differences between the two groups. Outcomes included Veterans RAND 12 (VR-12) physical component summary score (PCS), and PROMIS 29 domains for pain intensity, fatigue, pain interference, physical function, and sleep disturbance. Pruritus was assessed using the 4-D Itch scale for total itch. Multilevel, multiple linear regressions were used for outcome measures at 6 m post-injury. Random effects regression with robust standard errors (SE) were used to evaluate the impacts over time.

**Results:**

The study included 704 individuals and 92 were MDRO colonized (13%). Colonized patients had larger burns (25% TBSA, IQR 9-45 vs. 8% TBSA, IQR 3–20; p < .001), more operations (4, IQR 2-7 vs. 1, IQR 1-3; p < .001), more grafting (17% TBSA, IQR 3-46 vs. 3% TBSA, IQR 1- 9; p < .001), more ventilator days (2, IQR 0–8 vs. 0 IQR 0-0; p < .001), and longer LOS (34 days, IQR 17 – 64 vs. 16, IQR 9 - 27; p < .001). Adjusting for confounding covariables, such as demographics, colonization was associated with a lower PCS score (OR -0.33, 95% CI -0.68, -0.06; p=.018); a higher fatigue score (OR 0.46, 95% CI 0.13, 0.79; p = .007) and worse itch (OR 0.4, 95% CI -0.01, 0.75; p = .036). There was no association with pain intensity, pain interference, or sleep disturbance. Random effects regression indicated that colonization was associated with lower PCS (OR -5.0, 95% CI -8.60, -1.39; p = .007).

**Conclusions:**

Impact of colonization extends beyond the immediate hospitalization and likely has long-term effects on HRQoL. Given our observation of lower physical function after MDRO, more granular research on taxa-specific effects, timing of colonization, and interventions are indicated to elucidate the impact on HRQoL.

